# Leveraging Qualitative Insights for Dashboard Development to Address Perinatal Health Inequalities in Maternity, Neonatal and Perinatal Services

**DOI:** 10.1111/jep.70130

**Published:** 2025-05-22

**Authors:** Olufisayo Olakotan, Jennifer N. W. Lim, Mina Bhavsar, Farah Siddiqui, Rabina Ayaz, Gillian O'Brady Henry, Thillagavathie Pillay (Tilly)

**Affiliations:** ^1^ Department of Neonatology, Women and Children's Directorate University Hospitals Leicester NHS Trust Leicester UK; ^2^ Faculty of Education, Health and Wellbeing University of Wolverhampton Wolverhampton UK; ^3^ Leicester, Leicestershire and Rutland Local Maternity Systems Leicester UK; ^4^ Department of Obstetrics, Women and Children's Directorate University Hospitals Leicester NHS Trust Leicester UK; ^5^ Leicestershire Partnership NHS Trust Leicester UK; ^6^ Faculty of Science and Engineering University of Wolverhampton Wolverhampton UK; ^7^ Department of Population Health Sciences University of Leicester Leicester UK

**Keywords:** dashboard, evaluation, maternal and neonatal outcomes, perinatal health inequalities, quality improvement

## Abstract

**Introduction:**

Perinatal health inequalities, referring to disparities in maternal and neonatal outcomes among different ethnic groups, persist despite numerous calls to action. A quality improvement initiative, such as the perinatal health inequality dashboard, could serve as a tool to provide real‐time data, highlight trends and patterns, and capture and analyse disparities in maternal and neonatal outcomes across various population groups.

**Aim:**

To explore the development process of the perinatal health inequality dashboard and conduct a preliminary evaluation of the barriers and facilitators in effectively presenting data and highlighting disparities in maternal and neonatal outcomes on the dashboard in the Leicester, Leicestershire, and Rutland (LLR) region in the United Kingdom.

**Method:**

A qualitative study was conducted at the University Hospital of Leicester (UHL) involving the dashboard development team and end users, who are clinicians specializing in obstetrics, neonatal care, and perinatal mental health from the Leicester, Leicestershire, and Rutland (LLR) region. Ten clinicians were interviewed using a semi‐structured approach over a 3‐month period. The data were analysed using thematic analysis following Braun and Clarke's method.

**Results:**

The study findings are presented in two sections: the dashboard development process and dashboard evaluation. Key themes in the development process include data sourcing, integration, and accuracy in neonatal care. Evaluation themes focus on the potential impact of the dashboard, a user‐friendly interface, balancing qualitative and quantitative data, sustainability through continuous oversight, and system integration and interoperability. These findings offer critical insights for the ongoing refinement and effective deployment of the dashboard.

**Conclusion:**

The dashboard has the potential to improve health outcomes based on clinicians' insights. However, continuous refinement and modification of its functionality are necessary to address the challenges associated with its use.

## 1 Introduction

Perinatal health disparities exist globally, significantly impacting the most vulnerable and economically disadvantaged populations. These disparities, particularly pronounced among ethnic minorities, affect birthing individuals, fetuses, and newborns [1, 2, 3, 4, 5]. The 2018 report by Mothers and Babies: Reducing Risk through Audits and Confidential Enquiries across the United Kingdom (MBRRACE‐UK) highlights significant disparities in maternal mortality rates among different ethnic groups in the United Kingdom [6]. Mortality rates are four times higher for Black birthing individuals, twice as high for birthing individuals of mixed ethnicity, and nearly twice as high for Asian birthing individuals compared to White birthing individuals [6].

Findings from the Leicester, Leicestershire, and Rutland (LLR) region mirror these national trends, revealing adverse health outcomes in the most deprived areas, particularly among ethnic minority groups [7]. Data from the 2011 and 2019 censuses show that 72% of Leicester's population resides in the 40% most deprived areas, nationally nearly double the national average [7, 8]. High infant mortality rates in LLR, consistently among the highest in the United Kingdom, are closely linked to social deprivation and ethnicity [9]. Furthermore, ethnic minority women with mental health issues in LLR face greater barriers in accessing care compared to their White British counterparts [10]. Similar trends are observed in other developed economies. For example, in the United States, Canada, and Australia, Black mothers not only face higher maternal mortality rates than White mothers, but they also give birth in hospitals with higher risk‐adjusted rates of stillbirth and neonatal death [11, 12, 13].

One emerging solution is the use of health inequality dashboards, which enable the visualization and analysis of health data to identify disparities and facilitate targeted interventions [11, 14, 15, 16]. For example, the Ohio Children's Opportunity Index (OCOI) and Opportunity Index Dashboards (OID) enable healthcare professionals to analyse and compare inequalities [15, 16]. The dashboards help identify how structural factors such as racism, segregation, and social and economic conditions contribute to infant mortality in different states and cities [15, 16]. In Canada, a maternal‐newborn dashboard has heightened awareness of key indicators, guiding providers toward improved care and outcomes through behavioural changes and systematic methods, ultimately enhancing the patient experience [11].

Similarly, England introduced an inequality dashboard to track healthcare disparities within deprived neighbourhoods. Using eight indicators including patient‐to‐GP ratios and primary care quality, this tool focuses on areas with populations exceeding 100,000, visually presenting data to help decision‐makers address disparities [17]. Meanwhile, the California Perinatal Quality Care Collaborative (CPQCC) was designed to identify disparities in neonatal intensive care unit (NICU) outcomes and support targeted interventions to improve health equity for newborns and their families; however, its effectiveness is limited by its inability to fully capture deeper structural and interpersonal factors that contribute to inequities [18, 19]. These examples underscore the breadth of perinatal inequality dashboard approaches that drive data‐informed decision‐making and help reduce health disparities. The LLR perinatal health inequality dashboard draws on these global lessons while addressing region‐specific challenges. This study aims to explore the development process of the perinatal health inequality dashboard and conduct a preliminary evaluation of the barriers and facilitators in effectively presenting data and highlighting disparities in maternal and neonatal outcomes on the dashboard in the LLR region in the United Kingdom. The dashboard is currently in its mid‐development phase; thus, only a limited number of end users have had the opportunity to use or interact with it.

### 1.1 Description of the LLR Perinatal Health Inequality Dashboard

**Figure 1:**
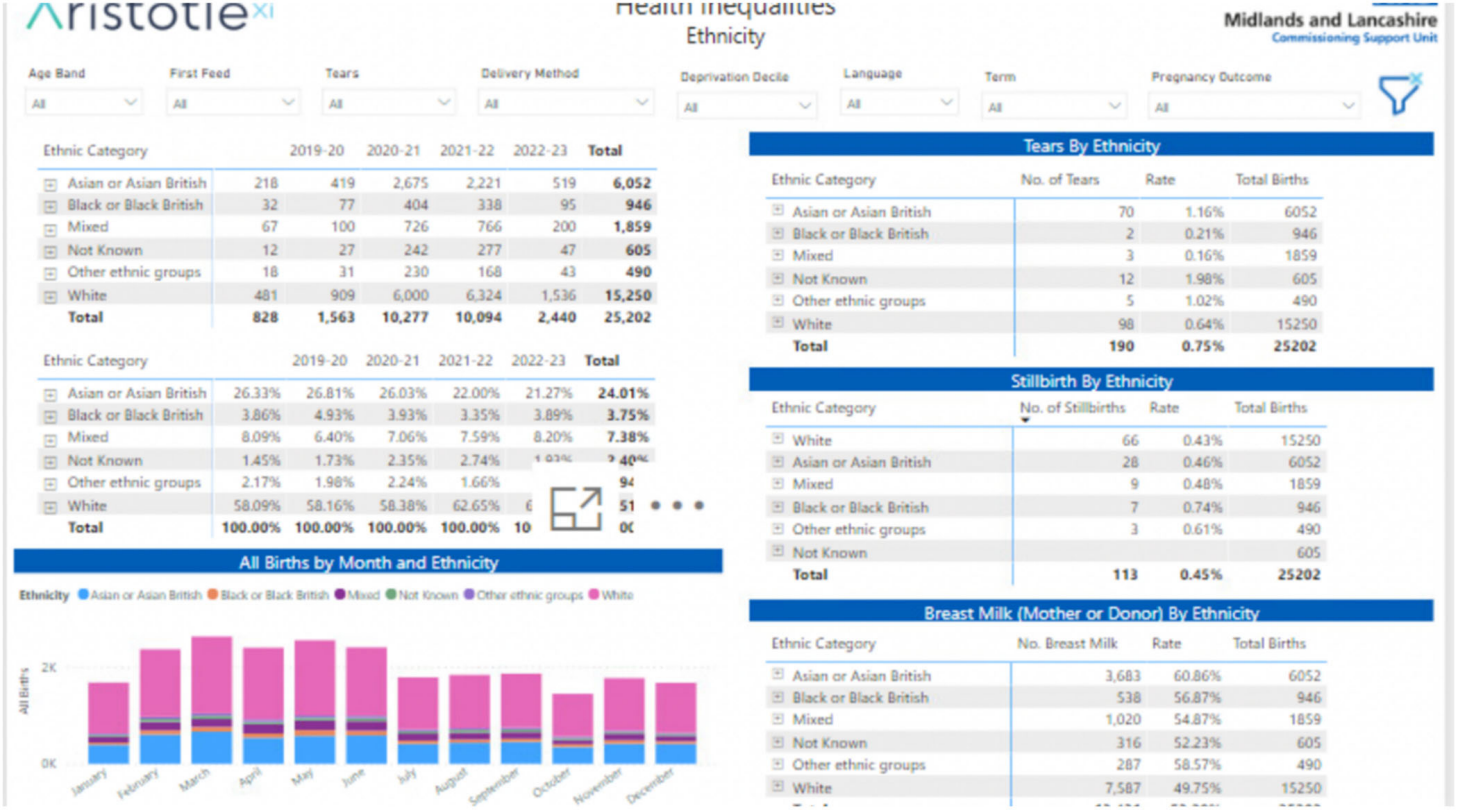
Sample of LLR perinatal health inequality dashboard.

The Local Maternity and Neonatal System (LMNS) is developing a perinatal health inequalities dashboard to address infant and maternal morbidity and mortality in the LLR region, with a specific focus on ethnic minority communities and areas of deprivation (Figure 1). The dashboard will report outcomes by ethnicity and language preference and identify factors contributing to disparities, including environmental factors, access to healthcare, patient‐related factors, and clinician‐related factors. Currently, the dashboard focuses on maternity and perinatal mental health, with future development planned to include a neonatal focus. The maternity pages include a summary statistics page and several additional pages addressing specific aspects, such as breastfeeding, deprivation factors, third‐degree tears, and birth outcomes. The Perinatal Mental Health section comprises two pages: one focuses on deprivation, and the other examines the relationship between ethnicity and factors such as age, preferred language, and source of referral.

### 1.2 Methodology

A qualitative study was conducted at Leicester Royal Infirmary (LRI), part of the University Hospitals of Leicester (UHL). The neonatal intensive care unit (NICU) at LRI provides level 3 (intensive) care, including care for neonates with surgical and complex cardiac needs. The service attends to approximately 10,000 births each year. Additionally, LRI serves as the base for the children's hospital, which provides treatment for various conditions associated with childbirth.

### 1.3 Participants

We employed convenience sampling to recruit ten participants, including clinicians specializing in obstetrics, neonatal care, and perinatal mental health; however, five individuals declined to participate. The participants were categorized into two distinct groups: developers, who were responsible for analyzing data and inputting it into the dashboard, and end users, who will interact with the dashboard on a daily basis. Integrating insights from both groups was essential to comprehensively understand the utility of the dashboard, ensuring it is both technically sound and practically effective.

The small sample size was limited by time constraints, as the dashboard was in its mid‐design and implementation stage, making developers and end‐users extremely busy and difficult to engage for recruitment and data collection. We acknowledge that theoretical saturation was not achieved due to the small sample size. However, the insights gained from this study provide valuable preliminary findings that contribute to perinatal health inequalities and lay a foundation for future research as the dashboard is further developed and implemented. The participants, aged 40–60 years, had 6–15 years of experience in their respective fields (Table 1). They were recruited based on their expertise and relevance to the study objectives. Ethnicity was not a criterion, and demographic data on race or ethnicity were not collected. This study adheres to the COREQ (Consolidated Criteria for Reporting Qualitative Research) guidelines, as detailed in Appendix A below [20].

**Table 1:** Demographics of participants.

Developers				
Participant ID	Clinical specializations	Age range (in years)	Experience (in years)	Gender
Dev 1	Obstetrics	45–50	6	Female
Dev 2	Neonatologist	40–45	8	Female
Dev 3	Neonatologist	50–55	10	Male
Dev 4	Obstetrics	45–50	7	Male
End users				
Ed 1	Perinatal psychologist	55–60	8	Female
Ed 2	Neonatologist	50–55	7	Female
Ed 3	Obstetrics and gynaecology	45–50	6	Female
Ed 4	Neonatologist	40–45	9	Male
Ed 5	Obstetrics	50–55	10	Male
Ed 6	Neonatologist	55–60	15	Female

### 1.4 Data Collection

Data were collected over 3 months by O.O., a clinical research fellow with training and expertise in qualitative research, using semi‐structured interviews with developers and end‐users. The interview guide was designed to gather perspectives on the dashboard's current and potential functionalities, given its early developmental stage [21]. Developers were asked questions about the technical aspects of the dashboard, while end‐users were asked evaluative questions regarding its potential applicability in addressing disparities in maternal and neonatal outcomes. The interviews, which lasted 45–60 min, were audio‐recorded and conducted both in person and virtually via Microsoft Teams.

### 1.5 Data Analysis

We employed the thematic analysis method by Braun and Clarke, which involves a six‐step approach: familiarization, generating initial codes, searching for themes, reviewing and defining themes, and producing the report [22]. Familiarization involved reading the transcripts multiple times and making informal notes. Coding was conducted by (O.O. and T.P.), who reviewed the transcripts to identify key themes and patterns related to the research question. The codes from all transcripts were then consolidated into broader themes and subthemes. A comprehensive review of these themes and sub‐themes was performed, resulting in the merging of relevant themes and the discarding of irrelevant ones. Finally, the synthesis of key findings was documented and reported. To ensure clarity and readability, participant quotes were lightly edited to remove filler words such as ‘um’, ‘you know’, and ‘like’, which do not alter the substantive meaning of their statements.

## 2 Results

A total of 10 clinicians, including developers and end‐users, were interviewed. The study findings are presented in two sections: the dashboard development process and dashboard evaluation. The development process revealed one key theme: data sourcing, integration, and accuracy in neonatal care. The evaluation addressed themes such as the potential impact of the dashboard, user‐friendly interface, key content for informed decision‐making, balancing qualitative and quantitative data, system interoperability and integration, and sustainability through continuous oversight and actionability. These themes provide insights into the practical challenges of developing a dashboard and how it should be designed to effectively highlight disparities in maternal and neonatal outcomes.

### 2.1 Dashboard Development Process

#### 2.1.1 Data Sourcing, Integration, and Accuracy in Neonatal Care

One of the developers highlighted that the dashboard integrates multi‐dimensional data types, encompassing neonatal, parental, and outcome data. They emphasized that this comprehensive data collection facilitates immediate clinical decision‐making and supports long‐term initiatives to address health disparities by identifying patterns in minority groups. For example: ‘We send about 40 data lines to the dashboard. We pull out information such as birth weight, birth gestation, and whether they are on breastmilk at discharge. We provide mothers' identifying details such as age, ethnicity, primary language, postcode, and GP's postcode; fathers' age, ethnicity, and occupation; whether they've had a high‐toxic insult; the number of levels of care days; and whether there's any brain injury. This allows us to look for patterns in minority groups and enhance our understanding of inequalities’. (Dev 1).

The dashboard's data is primarily sourced from neonatal databases, particularly *Badger Notes*—a system used within the NHS Trust that provides real‐time access to maternity, child, or neonatal records. One developer stated: ‘The Badger system remains our major source for drawing all relevant information related to maternal and neonatal outcomes’. (Dev 2).

However, despite the reliability of *Badger Notes* as a primary source, challenges with data accuracy persist due to data entry errors, especially during busy clinical shifts. Developers acknowledged that errors often arise when healthcare professionals input data under time pressures. One participant shared: ‘During a busy shift, data might be entered quickly, resulting in errors. During one shift, six babies were admitted between 7 and 10 o'clock. The junior doctor, due to go home after a 13‐hour shift, was trying to fill in data as quickly as possible’. (Dev 4).

To address these challenges, developers are integrating data from multiple systems (e.g., *Badger Notes* and the E3 system) to identify and rectify errors promptly. As one developer noted: ‘We sometimes have to glue the data together from different sources to make it work when data errors are discovered’. (Dev 3).

### 2.2 Dashboard Evaluation

#### 2.2.1 Potential Impact of Dashboard

End‐users cited several instances where the dashboard facilitated the examination of metrics such as third‐degree tears, breastfeeding, and perinatal mental health across various ethnic minority groups. They also noted that the dashboard aided in identifying disparities, trends, and patterns, such as increased complications in specific population groups, which might have been challenging to detect without this tool. For example, one end‐user explained, ‘It allows for comparisons because it tells you whether or not certain groups tend to have smaller babies. This dashboard enables you to see patterns and make comparisons, which means it's not just about how you feel in your practice when looking after women. Instead, you've got the information to back up your observations with evidence’ (Ed1). Another participant added, ‘With this tool, we can examine the third‐degree tears, breastfeeding, or perinatal mental health tab. It's about the interventions we can put in place and then finding the solution’ (Ed4).

### 2.3 Key Content for Informed Decision‐Making

End‐users emphasized that certain health‐related metrics, such as body mass index (BMI) and smoking status, are critical for informed clinical decision‐making. Several participants noted that the absence or insufficient emphasis on these data points in the current dashboard iteration limits its utility. For example, one end‐user expressed concern about the omission of key measures: ‘From the meetings I've been to recently on the dashboard, it doesn't say if a woman has a high BMI or if she smokes. I would like to see that on it, but I don't think that's going to be on it’. (Ed6). By including these metrics, the dashboard could better support clinicians in making evidence‐based decisions.

Another participant highlighted the challenge of managing information effectively while underscoring the importance of focusing on the most relevant metrics: ‘For me, the dashboard feels quite busy with a lot of information. While comprehensive data can be beneficial, prioritizing essential information is crucial to avoid the danger of excessive information’. (Ed1).

### 2.4 User‐Friendly Interface

Although they acknowledged the dashboard's potential to enhance clinical practice, end‐users stressed the importance of a more intuitive and visually distinct interface to mitigate information overload and highlight key disparities. One commonly suggested approach involved a traffic‐light colour‐coding system: red for alarming discrepancies, amber for minor deviations, and green for optimal results. For instance: ‘Dashboards should be well designed so that if there are discrepancies, say with a traffic light system, so if it's higher in the population for the Black and Minority Ethnic group, I'd like it to indicate that comparison, and it would be red and the real green.’ (Ed3).

Participants expressed a preference for Statistical Process Control (SPC) charts, highlighting their ability to present data trends in a clear, easy‐to‐interpret format. They contrasted SPC charts with simpler ‘rag ratings’, which they felt lacked depth and usability: ‘I think what I've asked to be included is more SPC charts, so that we can see things much more over a time span. Right now, it's more of a rag rating that doesn't provide depth of knowledge’. (Ed2). By presenting trends visually and clearly, SPC charts were seen to enhance the dashboard's user‐friendly interface, allowing clinicians to quickly interpret data and make informed decisions.

### 2.5 Balancing Qualitative and Quantitative Data

End‐users reported that the dashboard's reliance on quantitative data limits the understanding of cultural nuances and broader issues. One end‐user stated, ‘The dashboard provides numerical snapshots but misses important cultural contexts and individual narratives’ (Ed4). They advocated for the development of a platform or method to collect qualitative data, believing it would enhance numerical insights and contribute to a more comprehensive and informed interpretation. Another end‐user added, ‘We're not going to fully understand the reasons for not accessing services from a dashboard; we need qualitative data for that. It's about going out and speaking to people to get the full picture. Do they know about the service we provide, and would they be happy to be referred to a service like ours?’ (Ed3).

Furthermore, they suggested that to comprehend why breastfeeding rates are increasing or decreasing, factors such as changes in support services, including community midwifery, nursing, or health visitors, should be considered. One end‐user emphasized, ‘So, if it's breastfeeding, what are we looking at? Are we looking at improving the number of babies that are breastfed at a particular period of time? So, up to, say, six months? And so, if we're seeing that as a trend that the numbers are decreasing, I'd want to know why’ (Ed5).

### 2.6 System Interoperability and Integration

System interoperability and integration issues were identified as significant challenges, as end‐users noted that the dashboard operated in isolation and failed to communicate effectively with other systems, such as those used by health visitors and midwifery services. One end‐user stated, ‘There are different systems, such as Aristotle and the E3 system, in place for health visitors and midwifery services that don't necessarily communicate effectively with the dashboard’ (Ed4). Another stated, ‘We need these systems to talk to each other, and once the systems communicate effectively, it ensures seamless connections with other systems, and the dashboard functions optimally’ (Ed5).

### 2.7 Sustainability Through Continuous Oversight and Actionability

End‐users highlighted the importance of continuous monitoring, accurate analysis, and translating insights gleaned from the dashboard into actionable steps. One end‐user noted, ‘It requires consistent input and oversight. Someone needs to ensure the information is up‐to‐date and interpret the data to inform interventions’ (Ed1). Another stated, ‘Someone must oversee the dashboard and make interpretations from it. And that requires funding, time, and the ability to make interventions based on what we see’ (Ed4).

One developer stressed the importance of annotating the dashboard each time an intervention is implemented and monitoring its impact, noting that effective interventions should be sustained: ‘Mark on the dashboard that you've done an intervention. And then, so the dashboard is actually saying, on this day, to address this concern, we did this. And you hope to see a reduction in that concern that you've identified’ (D4).

## 3 Discussion

Our study findings demonstrate that the integration of comprehensive data types into the dashboard supports its potential to provide informative data for immediate and long‐term clinical decisions. Feedback from end‐users highlights how the dashboard should be designed to monitor maternal and neonatal outcomes and pinpoints further areas for improvement. One recurring sentiment is that end‐users are looking for ways to visually engage with complex statistics, especially when those figures have significant societal ramifications. This suggests that researchers, data scientists, and policymakers should consider the cognitive demands and preferences of their audience when conveying information about marginalized groups on the dashboard [15]. For stakeholders, investing in visual aids, infographics, and methods to streamline complex data into more digestible formats is a key strategy for designing interactive dashboards [23].

Involving stakeholders from different cultural backgrounds in the design and implementation of dashboards ensures that the dashboard remains useful, efficient, and effective [15, 16, 23, 24]. Proper stakeholder involvement is needed to ensure that the dashboard accurately captures the cultural nuances and challenges faced by different populations [15, 23]. Ensuring that the dashboard's language, images, and data presentation are culturally appropriate is necessary to identify factors contributing to perinatal health inequities, inform policy, and drive interventions.

Poor data quality is inherently linked to poorly documented information and a lack of rigor in data collection processes. Most healthcare organizations state that reporting race and ethnicity comes with both opportunities and challenges [25]. The opportunities include accurate reporting, which enables the identification of health disparities, informs targeted interventions, and supports policies aimed at achieving health equity [26]. However, challenges include inconsistencies in self‐reported data, potential misclassification, and the risk of reinforcing stereotypes if data is misinterpreted [26]. Additionally, it is important to acknowledge that race is a social construct that reflects lived experiences, systemic inequities, and social determinants of health. These factors ultimately influence health outcomes, but race itself should not be misinterpreted as a biological determinant of disparities.

Achieving success in data entry will depend on the commitment of diverse and cross‐functional teams; human involvement in this process often leads to errors [25, 27]. Additionally, while using multiple data sources can provide a holistic view and reveal disparities or patterns missed by a single data set, it also introduces challenges. Significant discrepancies in data collection methods can result in conflicting information and mislead users. Thus, addressing these issues requires careful consideration and standardization of data collection processes.

The growing preference for SPC charts among end users indicates a rising demand for analytical tools capable of providing deep insights into data trends over time. In healthcare, where quantitative measurements provide snapshots of different scenarios, understanding the contexts, trends, and underlying causes is essential. This is especially true when examining health inequalities. These insights are invaluable to development teams, as they guide the refinement of dashboards to meet user needs and expectations. By going beyond mere numbers to uncover deeper implications, a holistic perspective is achieved. This approach not only reveals underlying facts but also informs the decision‐making process aimed at reducing health disparities, thereby improving user satisfaction and engagement rates.

The importance of prioritizing interoperability and system integration cannot be understated, especially in perinatal care, where early intervention is crucial for the well‐being of birthing persons and children. Fragmented systems can pose significant obstacles [25]. Therefore, different systems serving health visitors and midwifery services should synergize so that relevant information can be extracted from the dashboard. This seamless integration would pave the way for real‐time insights and an in‐depth understanding of perinatal health inequalities.

### 3.1 Implication for Future Research

Future research should investigate how dashboards integrating multi‐dimensional data can be adapted and tested in diverse healthcare contexts, particularly in underserved or resource‐constrained settings. This includes exploring tailored approaches that account for variations in infrastructure, such as the availability of electronic health record systems, data‐sharing capabilities, and workforce capacity. Collaborating with stakeholders such as healthcare providers, policymakers, and community organizations will be critical to ensuring that implementation strategies align with local needs and capabilities.

To facilitate scalability, research must address practical resource and training requirements, including investments in technology, staff training, and ongoing support for end‐users. Studies should explore infrastructure enhancements, such as integrating dashboards with existing health information systems and ensuring interoperability across platforms. Developing automated mechanisms for data validation and real‐time feedback will also be essential for maintaining reliability and trust in the system.

Sustainability remains a key area for research, particularly in understanding how dashboards can be incorporated into routine workflows without overburdening existing systems. Efforts to evaluate funding models, ongoing oversight, and measurable outcomes from implemented interventions will ensure the long‐term impact of these tools. By addressing these practical considerations, future research can expand the utility of dashboards as scalable, transformative tools for equitable healthcare delivery.

### 3.2 Study Strength and Limitation

Feedback from end‐users and developers is crucial for shaping the current and future deployment of the perinatal health inequality dashboards, enhancing their potential as pivotal tools in reducing disparities through innovation and impact. However, as the dashboard is not yet fully functional and many end‐users have had limited interaction with it, the study results may present a biased view of its functionality. We acknowledge that theoretical saturation was not achieved in this study due to the small sample size, limiting our ability to capture the full range of perspectives. Larger samples will be essential to expand evaluation findings, ensuring that the dashboard can effectively contribute to reducing health inequalities in maternal and neonatal outcomes.

## 4 Conclusion

The study findings indicate that, for the dashboard to provide timely and relevant data to aid decision‐making, user‐centred design principles must be incorporated. Additionally, the dashboard should integrate effectively with other systems to enable seamless communication. Continuous and regular feedback from end‐users is essential to ensure that the dashboard remains a valuable tool in addressing disparities in maternal and neonatal care.

## Conflicts of Interest

The authors declare no conflicts of interest.

## Data Availability

The data sets generated and analysed during the current study are not publicly available due to confidentiality but are available from the corresponding author on reasonable request and pending ethics approval from UHL, NHS trust.

## Appendix A COREQ Checklist

**Table:**

Item no.	Guide questions/description	Reported on page #
Domain 1: Research team and reflexivity		
Personal characteristics		
1. Interviewer/facilitator	Which author/s conducted the interview or focus group?	5
2. Credentials	What were the researcher's credentials? for example, PhD, MD	5
3. Occupation	What was their occupation at the time of the study?	5
4. Gender	Was the researcher male or female?	N/A
5. Experience and training	What experience or training did the researcher have?	5
Relationship with participants		
6. Relationship established	Was a relationship established before study commencement?	N/A
7. Participant knowledge of the interviewer	What did the participants know about the researcher? for example, personal goals, reasons for doing the research?	5
8. Interviewer characteristics	What characteristics were reported about the interviewer/facilitator? for example, Bias, assumptions, reasons and interests in the research topic	5
Domain 2: study design		
Theoretical framework		
9. Methodological orientation and theory	What methodological orientation was stated to underpin the study? For example, grounded theory, discourse analysis, ethnography, phenomenology, content analysis	6
Participant selection		
10. Sampling	How were participants selected? for example, purposive, convenience, consecutive, snowball	5
11. Method of approach	How were participants approached? for example, face‐to‐face, telephone, mail, email	5
12. Sample size	How many participants were in the study?	5
13. Nonparticipation Setting	How many people refused to participate or dropped out? Reasons?	5
14. Setting of data collection	Where was the data collected? for example, home, clinic, workplace	5
15. Presence of nonparticipants	Was anyone else present besides the participants and researchers?	N/A
16. Description of sample	What are the important characteristics of the sample? for example demographic data, date	6
Data collection		
17. Interview guide	Were questions, prompts, and guides provided by the authors? Was it pilot tested?	5
18. Repeat interviews	Were repeat interviews carried out? If yes, how many?	N/A
19. Audio/visual recording	Did the research use audio or visual recording to collect the data?	5
20. Field notes	Were field notes made during and/or after the interview or focus group?	N/A
21. Duration	What was the duration of the interviews or focus group?	5
22. Data saturation	Was data saturation discussed?	5
23. Transcripts returned	Were transcripts returned to participants for comment and/or correction?	N/A
Domain 3: analysis and findings		
Data analysis		
24. Number of data coders	How many data coders coded the data?	6
25. Description of the coding tree	Did the authors provide a description of the coding tree?	N/A
26. Derivation of themes	Were themes identified in advance or derived from the data?	6
27. Software	What software, if applicable, was used to manage the data?	N/A
28. Participant checking	Did participants provide feedback on the findings?	N/A
Reporting		
29. Quotations presented	Were participant quotations presented to illustrate the themes/findings? Was each quotation identified? for example, participant number	7–9
30. Data and findings consistent	Was there consistency between the data presented and the findings?	7–9
31. Clarity of major themes	Were major themes clearly presented in the findings?	7–9
32. Clarity of minor themes	Is there a description of diverse cases or a discussion of minor themes?	7–9
